# The social impact of COVID‐19 as perceived by the employees of a UK mental health service

**DOI:** 10.1111/inm.12883

**Published:** 2021-05-21

**Authors:** Clare M. Eddy

**Affiliations:** ^1^ Birmingham and Solihull Mental Health NHS Foundation Trust and College of Medical and Dental Sciences University of Birmingham Birmingham UK

**Keywords:** coronavirus, interpersonal relations, nurse–patient relations, personal protective equipment, physician–patient relations

## Abstract

This study explored the perceptions of NHS employees working within a UK mental health trust in relation to the social impacts of the COVID‐19 pandemic. Questioning focussed on social isolation and desire to interact with others before and since COVID‐19; effects of safety measures including personal protective equipment and social distancing; and perceived influences of the pandemic on service users and social aspects of service delivery. All employees at an English NHS mental health service were invited to complete an anonymous online questionnaire (July–September 2020), resulting in 464 completed questionnaires. Response frequencies were summed across the total sample, and the influence of patient contact, age, and vulnerability to COVID‐19 were explored using pairwise comparisons. Approximately two thirds of employees felt there had been a fundamental change in how they felt about interacting with others, and many had lost confidence in their ability to relate emotionally to others. Respondents were keen to adhere to safety guidance, but the majority believed that face masks and social distancing could have a detrimental effect on communication and rapport within the workplace. Other concerns included passing on the virus, social isolation of employees and service users, and a reduction in community services. COVID‐19 safety measures may impact morale, communication, empathy, and the provision of client‐centred care. More generally, the pandemic has changed the attitudes of mental health workers towards social interaction, with younger employees reporting more mental health difficulties that may be linked to concerns about longer term social change.

## Introduction

COVID‐19 was classified as a pandemic by the WHO in March 2020. Beyond the potential threat to physical health, there are growing concerns over the impact of the COVID‐19 pandemic on mental health (Cao *et al*. 2020; Gruber *et al*. 2020; Pfefferbaum & North 2020). In addition to advocating the use of personal protective equipment (PPE), governments across the globe have enforced measures including physical (or social) distancing between people. These changes have speeded a revolution in the operation of health services, whereby most outpatient care is now delivered virtually. However, a loss of in‐person human contact may pose drawbacks (Haider *et al*. 2020). Emerging reports suggest that loss of social interaction associated with the pandemic may lead to feelings of alienation and loss of self‐worth (Williams *et al*. 2020), and that physical distancing can impact individual well‐being (Haider *et al*. 2020) across the lifespan (Beam & Kim 2020; Flint *et al*. 2020).

Adequate social contact is critical for mental health (Hawkley & Cacioppo 2010; Miller 2011). Therefore, perhaps the impact of COVID‐19 on social behaviour and related psychological well‐being could not be of greater relevance than to mental health services. Service users have existing psychological vulnerabilities, and experience lifestyle difficulties which mean the pandemic may exert increased adverse effect (Kaufman *et al*. 2020), and healthcare workers employed within mental health services experience significant exposure to psychological stressors (Rimmer 2018). Research exploring the effects of the pandemic on both mental health workers and service users will therefore be of great value.

## Background

Recent studies have shown that the pandemic has exacerbated mental health difficulties in general (Duan & Zhu 2020; Liebrenz *et al*. 2020) leading to greater pressure on mental health services worldwide. There is also evidence that healthcare workers may be particularly vulnerable to psychological problems as a result of COVID‐19 (Huang & Zhao 2020; Rossi *et al*. 2020). However, while numerous previous studies have explored the effect of the pandemic on healthcare workers’ psychological well‐being, few have focussed specifically on mental health services, explored attitudes towards safety measures within the workplace, or examined the social impact of the pandemic within the workplace or more widely.

Anxiety around catching the virus is likely to have impacted individual attitudes towards social interaction. In addition, where closer contact with others may be desired or beneficial (e.g. in order to show empathy), the ability to engage in this may be restricted by safety measures such as social distancing and the use of face masks (Wong *et al* 2013). Asides from physical contact, both verbal and non‐verbal communication are other critical aspects of social interaction that may be detrimentally affected by the use of personal protective equipment (PPE). Although there will be a considerable impact in wider society, the social effects of the pandemic are highly relevant to mental health services, given that clinician–patient relationships and wider social support critically influence intervention and therapeutic alliance (Brown *et al*. 2014). For example, previous studies have shown that clinician facial expressions can influence service user understanding, engagement, and treatment success (Ambady *et al*. 2002). It is important to understand how the pandemic has influenced social interaction between mental health service users and employees, and how any related workplace changes may have detracted from the ability to provide effective care.

The current study explored the social effects of the COVID‐19 pandemic, within the workplace and more generally, as perceived by employees working in a UK mental health Trust. The pandemic (virus and related safety measures) could influence an individual's perspectives about other people, desires pertaining to social interaction, workplace rapport, empathy, and communication. Previous studies have shown that attitudes towards a pandemic and related safety measures may vary over time according to the settings in question, demographics, and psychological factors (Bish & Michie 2010; Kaspar 2020; Taylor *et al*. 2020). Therefore, the opinions of those employees experiencing direct contact with patients were compared to those employees who do not, and the perceptions of those participants who were older/vulnerable to COVID‐19 were compared to those who were younger and less vulnerable. The aim was to highlight issues worthy of further research relevant to mental health employees and service users, with a view to informing service delivery.

## Methods

The study was approved by the host NHS Research Department and the Health Research Authority (UK). Participants were recruited from a single Mental Health Trust in England, which provides both inpatient and outpatient services, including secure care, community outreach, and specialist services (e.g. neurological/sensory impairments).

All Trust employees (approximately 4000) were invited to take part in an anonymous online survey through intranet adverts and Trust‐wide email between late July and late September 2020. After informed consent, the next 9 survey questions collected basic demographical information (e.g. job role, age, vulnerability to COVID‐19, results of COVID‐19 tests). Participants were then asked to read each statement that followed and decide how much they agreed with it, answering using a 5‐point Likert scale (see Table 1). Some statements were worded negatively to control for response bias. The questionnaire was developed by the author, pilot tested within the Trust Research and Innovation Department to assess face validity, and the scale and wording of questions was modified after feedback from colleagues to improve scope and understanding. Question context varied according to people involved (e.g. close others, work colleagues, service users, strangers), place (inside or outside of the workplace), and timescale (e.g. now, future). Twenty questions explored habits and feelings about social interaction in general, and how these may have changed during the pandemic. Next, there were seven questions about perceived risks relating to COVID‐19. The third section (12 questions) asked about the perceived social impact of COVID‐19. The following section (18 questions) focussed on COVID‐19 related issues within the working environment. Participants were able to decline to answer specific questions, and where relevant, a not applicable option was included (e.g. for respondents not working in a patient facing role). Finally, a non‐compulsory general feedback option was included.

**Table Table 1:** Social impact of COVID‐19 questionnaire

Questionnaire section	Example statements^†^
Habits and feelings related to social interaction in general	I think COVID‐19 has negatively affected the quality of my relationships… I am keen to stick to the recommended social distancing policies… I prefer/red to meet up in person… I find it fairly easy to feel close to other people emotionally…
Risks related to the pandemic	I am concerned that I will catch COVID‐19… I think that being in the workplace leads to a significant increase in my risk… I am not concerned that I could pass on COVID‐19… I feel the COVID‐19 situation has affected my mental health…
Social impact of COVID‐19	I talk to people less often overall since COVID‐19… Changes made due to COVID‐19 have made me realize how important physical contact can be… COVID‐19 has not fundamentally affected the way I feel about interacting… I am concerned about the long term social effects of COVID‐19…
Impact of COVID‐19 within the working environment	I do not think COVID‐19 measures negatively affect social cohesion… I think that face masks can adversely affect communication … I think face mask health benefits always outweigh the negative social effects… I do not think COVID‐19 has made service users feel more socially isolated… I do not think the effects of social distancing can be a barrier to expressing empathy… There are certain social activities I would prefer not to undertake now with patients…

^†^ Responded to using a 5‐point Likert scale, from strongly agree to strongly disagree.

All responses were included in analysis. First, response frequencies were determined for each question for the whole sample. Next, Mann–Whitney *U*‐tests were used to compare subgroups according to age, patient contact, frequency of home working, and vulnerability to COVID‐19. Findings surviving Bonferroni correction are indicated*. Finally, themes arising from feedback comments were summarized. Themes were identified by summing common words/phrases contained within the free response field, across the 67 participants who chose to complete this optional section.

## Results

### Sample characteristics

A total of 464 Trust employees completed the questionnaire. Most respondents were in the 45‐ to 55‐year‐old age bracket (36%) followed by 36–45 (21%), 26‐35 (19%), 56–65 (19%), 18–25 (4%), and >65 (1%). There was good representation across different service areas (acute care: 9.5%; secure care: 18%; corporate: 27%; community: 29%; specialties: 16%). Slightly more employees worked within a clinical role (56%) and a good majority (77%) experienced at least occasional contact with patients. Similar numbers of respondents often could, sometimes could, and rarely could, work from home (32; 31; 36%). Just over half of respondents (54%) thought they had not had COVID‐19, and 19% thought it was likely they had. However, only 3% of respondents had tested positive, although 13% had received a positive antibody test. One third of respondents reported that they were vulnerable to COVID‐19 due to age, ethnicity, or medical conditions.

### Response frequencies

For ease of interpretation, responses were combined where respondents agreed/strongly agreed, and disagreed/strongly disagreed. Strongly polarized results are indicated (SA: strongly agree).

#### Thoughts on social interaction

While 82% of respondents preferred meeting well known people in person before the pandemic (47% SA), this dropped to 44% at the time of questioning, but 63% of employees expected to do this in the future. Preference to meet less well known people fell from 42%, to 26%, and 27% in the future. When questioned, 71% of respondents were keen to stick to social distancing guidelines around close others (31% SA), 84% around colleagues, 89% (60% SA) around service users, and 95% (75% SA) around strangers. Only 53% expressed a preference to maintain this with close others, but more wanted to maintain this with colleagues (71%), patients (78%), and strangers (89%; 60% SA).

Many respondents (44%) believed the pandemic has had a negative effect on their relationships with close others (32% expected this to last), and even more (55%) with less close others (49% expected this to last). Before the pandemic 76% of respondents were confident they could relate well to others emotionally, but only 45% agreed with this sentiment post‐pandemic. Almost four times as many respondents felt they could not relate well emotionally to others post‐pandemic.

#### Feelings about COVID‐19

Responses indicated that 61% of respondents were concerned about catching the virus and just 11% were not concerned. More than two‐thirds (67%) felt that being in the workplace (despite safety measures) increased their risk. However, more employees were concerned about their close others catching the virus (88%), and 78% were concerned about passing it on to close others. 56% of respondents believed that the pandemic had affected their mental health, and most affected in this way (45% of total sample) believed that this would continue. Only 30% of respondents were feeling less worried about COVID‐19 with time.

#### Social effects of the pandemic

Despite the availability of technology, 56% of respondents believed that they talk to other people less overall since the pandemic and 66% felt more socially isolated. The vast majority of respondents had realized the importance of physical contact with close others (88%; 46% SA) and with service users (74%). A very high number of employees were concerned about the longer term impacts of the virus (84%) and of related measures (e.g. PPE, social distancing: 85%).

For 55% of respondents, feelings about interacting with close others had fundamentally changed as a result of the pandemic. A similar amount of employees felt this way in relation to service users (59%) and colleagues (56%), and even more with strangers (73%). In relation to the general desire to interact with others, 45% reported a reduction towards close others, and 62% a reduction towards less close others.

#### Specific effects of COVID‐19 and related measures in the workplace

In the workplace, measures such as PPE and social distancing were thought by 80% of respondents to affect the well‐being of employees, and 78% believed they could impact service user well‐being. Furthermore, 63% of respondents agreed or strongly agreed that social cohesion between employees and patients had been affected, and 39% agreed (37% disagreed) that the ability to attend to the social needs of patients had been adversely impacted. Many respondents thought face masks could negatively impact interaction with colleagues (62%) and service users (65%), making communication more difficult with service users (77%) perhaps slightly more so than with colleagues (66%). While most respondents believed that the potential benefits of face masks tended to outweigh the costs (62%; 21% undecided), many felt they were not a favourable long term option (50%; 25% undecided).

A rise in mental health problems was identified by 82% of participants. A very high number of employees also thought service users had become more socially isolated (83%) and had concerns about support for them in the community (87%). Many respondents agreed that safety measures could be a barrier to empathy (44%) including social distancing (45%). More specifically, 42% of respondents agreed or strongly agreed that social distancing could restrict their ability to show empathy towards service users (22% undecided). In addition, there were certain activities 75% of employees would rather not undertake with service users since the pandemic, and this was the case for 49% even when PPE was available.

### Subgroup comparisons

#### Respondents self‐identifying as vulnerable to COVID‐19 (*n* = 310) versus those not vulnerable (*n* = 154)

Sixteen questions relating to risk and desire to interact with others were compared for the vulnerable and non‐vulnerable subgroups (Fig. 1). Those within the vulnerable category believed more that: they had become more socially isolated (MWU = 20628, *P *= 0.012); their mental health was currently affected (MWU = 21079, *P* = 0.032); they might catch the virus (MWU = 18842, *P* < 0.0001*); being within the workplace was leading to greater risk (MWU = 19223.5, *P* < 0.0001*); and they would prefer not to undertake social activities with patients despite PPE being available (MWU = 9916, *P *= 0.002).

**Fig. Fig. 1:**
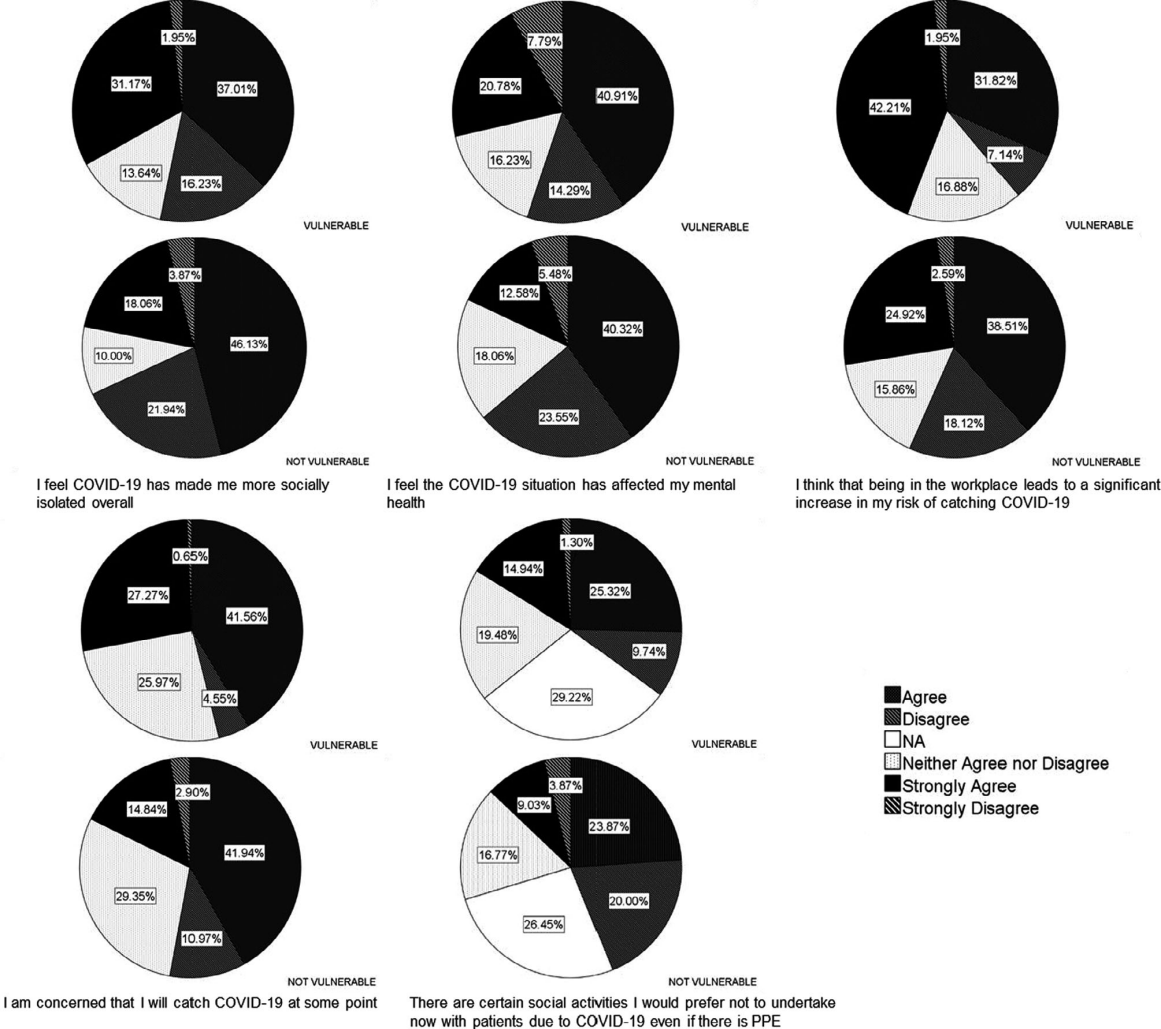
Response frequencies according to vulnerability to COVID‐19.

#### Relatively younger (aged 18–35: *n* = 108) compared to relatively older (aged 56+; *n* = 93) respondents

Sixteen questions relating to social distancing, risks, mental health, and desire to interact with others were compared for younger versus older respondents (Fig. 2). Younger respondents were less keen to social distance from close others (now: MWU = 4101, *P *= 0.018; future: MWU = 3720, *P* < 0.001*); were becoming less concerned with catching COVID‐19 over time (MWU = 3540, *P* < 0.0001*); agreed more that the pandemic had affected their mental health (MWU = 3433, *P* < 0.0001*); were more likely to be concerned about the long term effects of safety measures (MWU = 4159, *P *= 0.022); and disagreed more that they were not concerned about passing the virus on to close others (MWU = 4092, *P *= 0.021).

**Fig. Fig. 2:**
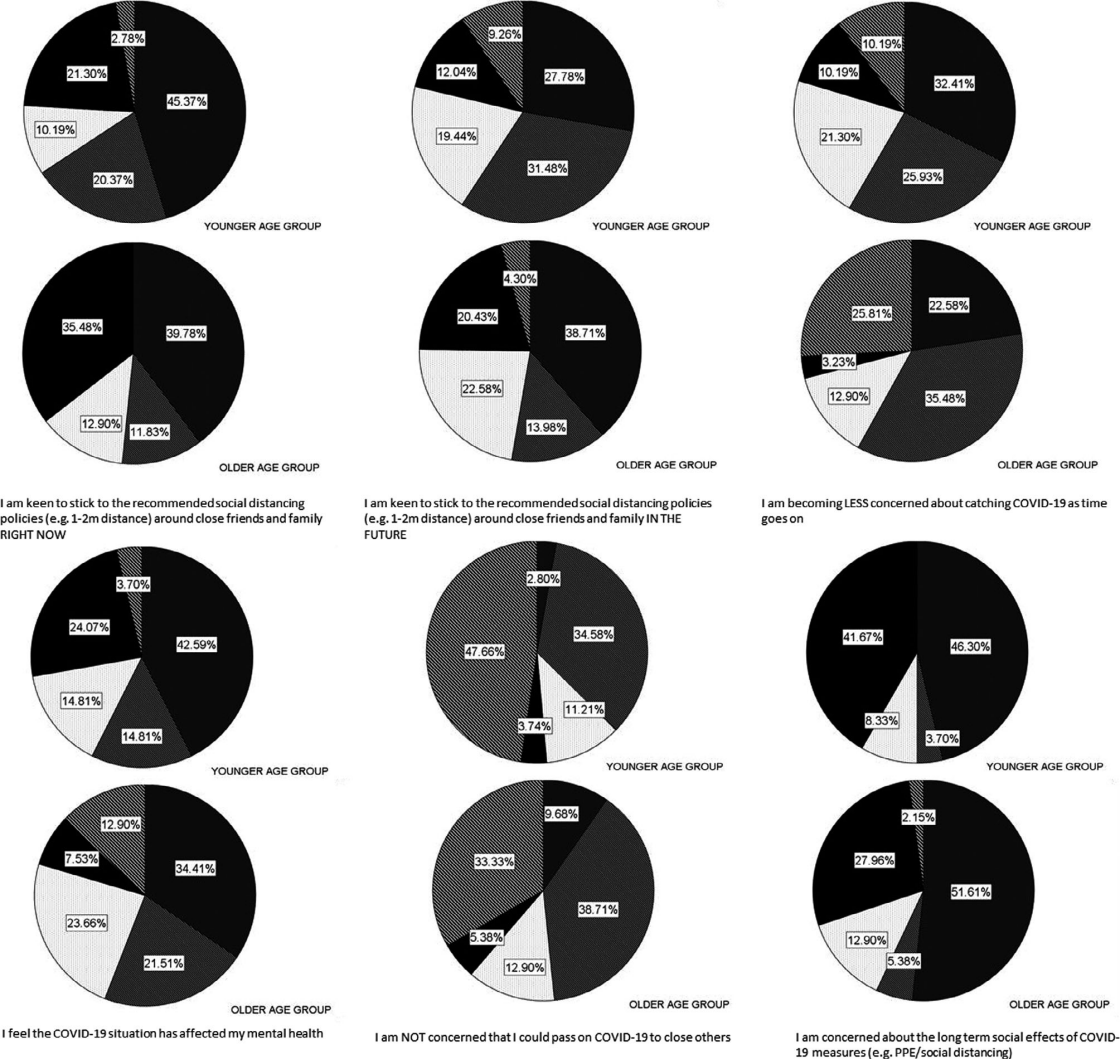
Response frequencies according to younger (18–35) or older (56+) age group.

#### Respondents in patient contact roles (*n* = 357) versus those in roles without patient contact (*n* = 107)

Employees reporting at least occasional patient contact as part of their role were compared to those reporting no patient contact for 14 questions relating to service users (Fig. S1). For many questions, the not applicable option was selected (by many participants in the no patient contact group). Those employees who experienced patient contact agreed more that: some practices related to COVID‐19 negatively impacted patient well‐being (MWU = 9682, 348 vs. 69; *P *= 0.006); face masks negatively impacted interaction with service users (MWU = 7580, 344 vs. 54; *P *= 0.021); service users had become more social isolated since COVID‐19 (MWU = 8287.5, 343 vs. 64; *P *= 0.001*); social distancing was a barrier to expressing empathy (MWU = 15213, 356 vs. 107; *P *= 0.001*); and they would prefer to avoid certain social activities with patients if PPE was not available (MWU = 4682, 302 vs. 39; *P *= 0.026).

#### Respondents who could often/usually work from home (*n* = 150) compared to those who could rarely/never work from home (*n* = 169)

Sixteen questions relating to social distancing, mental health, feelings about catching the virus, social isolation, and desire to interact were compared for the subgroups who could often work from home and those who could never/rarely work from home (Fig. S2). Frequent home workers were more keen to stick to social distancing guidelines with colleagues (now: MWU = 8470.5, *P* < 0.0001*; future: MWU = 8909.5, *P* < 0.0001*), service users (now: MWU = 10461.5, *P *= 0.002; future: MWU = 9993, *P* < 0.0001*), and strangers (now: MWU = 10941, *P *= 0.007; future: MWU = 10955, *P *= 0.017).

### Themes arising from feedback comments

Sixty‐seven individuals provided feedback. A few people commented that the negative wording of some questions could be confusing (although this was necessary to help avoid response bias), but the vast majority commented on their work‐related experience of the pandemic (Table 2).

**Table Table 2:** Themes arising from feedback comments

Theme	Examples
General negative effects of the pandemic	Loneliness Greater anxiety around using public transport Communication difficulties Increased staff absence and burnout Feeling uncertain or unsafe
General positive effects of the pandemic	Working from home meaning more family time Avoidance of a stressful commute Enhanced communication and support from management and colleagues Greater appreciation of relationships with others
Attitudes towards COVID‐19 related measures	Feeling inhibited by restrictions while sometimes observing that other staff were more relaxed Friction between staff as a result of perceived variations in adherence to guidance A need for more training around the use of PPE Believing there is a need for increased flexibility and responsiveness as measures sometimes appear disproportionate to actual risk
Perceived negative impacts on staff experience of service delivery	A reduction in multi‐disciplinary team meetings Communication difficulties due to not being able to read facial expressions Distraction, discomfort and deterioration in morale due to wearing a face mask Difficulties expressing empathy towards patients while maintaining social distancing
Perceived negative impacts on service user experience of service delivery	A detrimental impact on patient clinician interactions because additional safety measures have made the carrying out of personal health interventions less sensitive and person‐centred Potentially unnecessary restrictions around social activities leading to boredom and frustration Greater reliance on Trust staff as a primary source of social support Increased anxiety due to poor provision of Primary Care and other external services

## Discussion

The COVID‐19 pandemic has influenced the perceptions and attitudes of mental health trust employees in relation to social interaction. Roughly half of respondents agreed they had experienced a general reduction in the desire to socialize with others since the pandemic, in line with findings from a previous study (Williams *et al*. 2020). Preferences around meeting people in person have reduced, with the most long lasting impact on the desire to meet less well known others in future. How well respondents knew the other people in question had a linear relationship with the desire to maintain social distancing, with motivations to maintain distancing being highest around those least well known. However, more than half of employees expected to maintain social distancing with close others in the future. Frequent home workers were more keen to stick to social distancing guidelines with colleagues, service users, and strangers. This could reflect more of those working from home being in the vulnerable category, or that working from home could encourage more fear about the risks of social contact. Overall, mental health employees were highly motivated to follow social distancing rules, in accordance with generally high compliance within the UK (Williams *et al*. 2020). Further research is needed to understand how attitudes may vary due to psychological (e.g. fear of catching/spreading the virus; social disapproval; mental health; etc.) and environmental (e.g. availability of PPE; occupational practicalities) factors.

Many respondents felt the pandemic had negatively impacted their relationships with others, including close others. This could reflect psychological closeness, provision of practical support, differing opinions about COVID‐19, or other influences. More specifically, a significant proportion of employees (approx. 31%) felt they were less able to relate to others emotionally since the pandemic. Experiencing stress can reduce the capacity to empathize (Martin *et al*. 2015; Park *et al*. 2015), so this finding could reflect external factors such as social distancing or mental health issues. The ability to give and receive emotional support is crucial within health services, so these findings will be important to follow up.

Concerns about contracting the virus within the workplace remained high (particularly for vulnerable respondents), despite active safety measures. Employees were particularly concerned about passing COVID‐19 on to others. Over half of respondents reported a reduction in communication with others or social isolation, including those still attending the workplace. Worry about passing on the virus could have even greater impact on mental health than simply catching it, especially in younger people. Younger respondents (aged 18–35 years; vs. those aged 56+) were less keen to socially distance from close others, but were also more concerned about passing on the virus. This demonstrates dissonance in terms of desire to be close to loved ones, despite an awareness of the greater vulnerability of older people to COVID‐19. Younger respondents were also more likely to report mental health difficulties and were more likely to be concerned about the long term effects of safety measures such as PPE and social distancing. For younger healthcare workers, mental health effects associated with the pandemic therefore surpass fears about vulnerability to the virus and may arise through the impact of wider societal and economic changes.

A very high proportion (78; 80%) of respondents thought that safety measures could affect the well‐being of service users and employees, and many agreed that a reduction in social cohesion may be one result. Two thirds of employees (especially those in patient facing roles) felt that face masks impaired communication with colleagues and service users, with the latter affected to a greater extent. Indeed, at least one published study supports the possibility of impaired communication in association with PPE (Hampton *et al*. 2020). The risks associated with impaired communication are particularly high in clinical settings, especially when working with populations experiencing primary communication difficulties. Use of PPE by both patients and treating clinicians may affect communications influencing diagnosis and treatment.

Mental health trust employees believed there had been an increase in mental health problems since the pandemic and the vast majority believed that service users were experiencing social isolation and a reduction in community support. Empathy, which is essential to the therapeutic process (Jani *et al*. 2012), was thought to have been impaired by up to half of respondents, sometimes hampered by the use of safety measures. Confidence at undertaking certain (unspecified) activities with service users had also reduced, and concerns were not always ameliorated by the provision of PPE.

Further research is needed given that we do not fully understand how healthcare workers’ responses may have been shaped by factors such as personality (Carvalho *et al*. 2020), income/socio‐economic status (Weill *et al*. 2020), self‐efficacy, and health status (Kaspar 2020; Oosterhoff *et al*. 2020). Other limitations associated with the current study include the use of a non‐validated questionnaire and that information about gender was not collected, although male gender could pose additional vulnerability to COVID‐19 mortality (Kelada *et al*. 2020). Future related research would be enriched through inclusion of more in‐depth qualitative methods and should aim to verify the importance of the themes identified in this study. Finally, further development of the questionnaire used including checks for reliability and validity would strengthen future findings.

## Conclusions

In conclusion, the pandemic has led to fundamental changes in the social attitudes and perceptions of UK NHS mental health trust employees towards close and less close others, inside and outside of the workplace. Concerns about the risks associated with social interaction at work were not always abated despite the adoption of safety practices, and specific worries about transmission to close others remain. Employee morale and confidence in the ability to provide care to service users in the ways they aspire to (i.e. with empathy, effective communication, integrated with external services, and adopting a personalized approach) have also been significantly impacted by COVID‐19 and related measures. Younger employees may be more psychologically affected despite being less physically vulnerable and are more concerned about the longer term social impact of safety measures. It is hoped that these initial findings will be invaluable in informing further research that will help service providers understand how best to encourage adherence to recommendations while effectively supporting both the mental and physical well‐being of their employees and service users.

## Relevance to Clinical Practice

This study has shown that prolonged home working is associated with greater anxiety around social interaction post‐pandemic. However, even employees regularly attending the workplace talk to each other less and report feelings of social isolation. In addition to monitoring employee well‐being, employers should encourage a sense of social cohesion and support the expression of empathy between individuals. Employees should return to the workplace whenever possible and safe opportunities for employees and service users to interact without the use of PPE should be identified and facilitated.

## Funding

This study was unfunded.

## Data Availability Statement

Data can be provided by the author (clare.eddy1@nhs.net) in response to reasonable requests received within three years following study publication.

## Supporting information


**Figure S1**. Response frequencies according to whether job role involves patient contact.
**Figure S2**. Response frequencies according to whether respondents can usually work from home or can never/rarely work from home.
