# Supplementary material for: The social impact of COVID‐19 as perceived by the employees of a UK mental health service
Source: Int J Ment Health Nurs. 2021 May 21;30(Suppl 1):1366–75. doi: 10.1111/inm.12883 (PMC8242788; doi:10.1111/inm.12883)

**
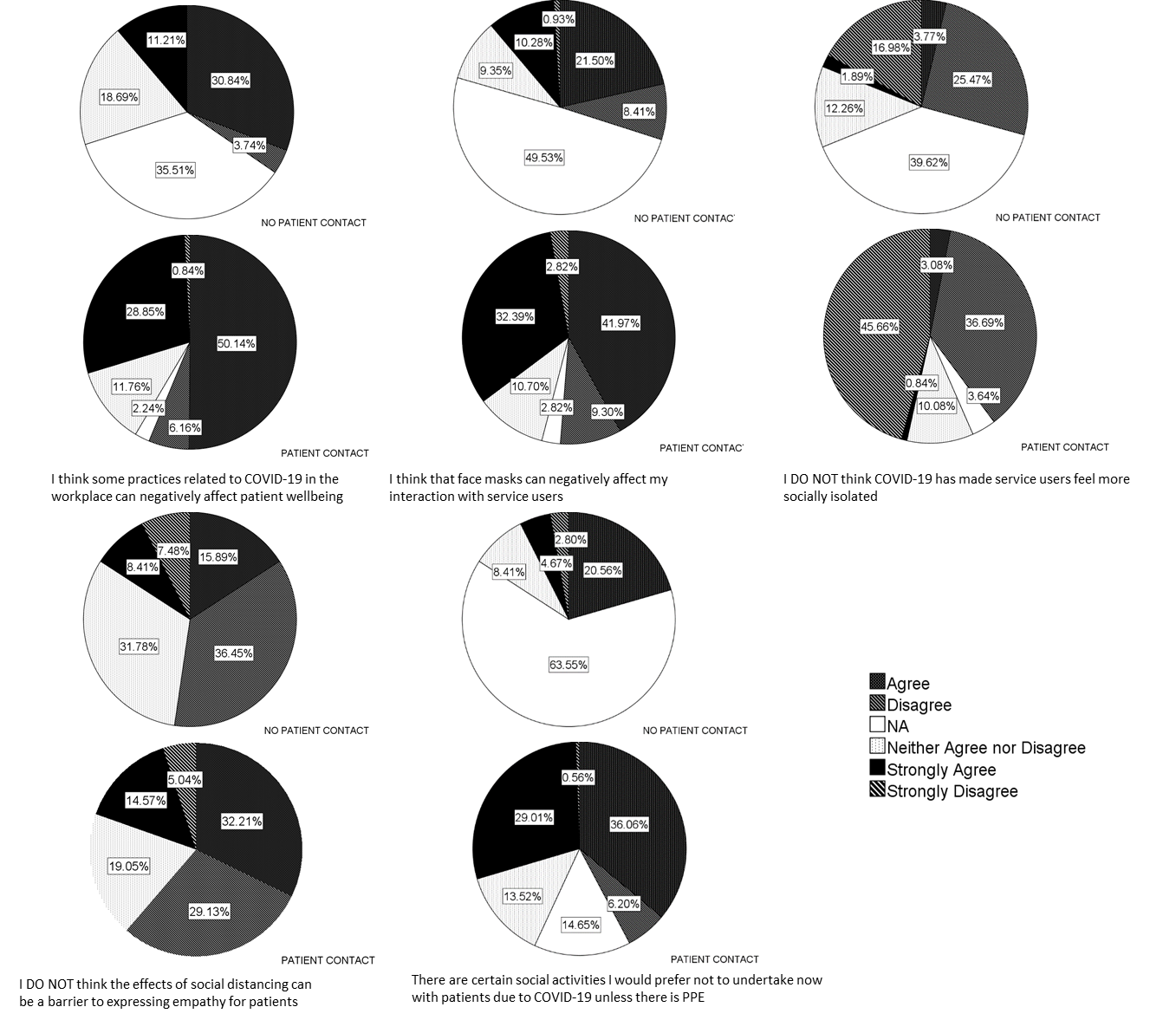
Supplementary Figure 1. Response frequencies according to whether job role involves patient contact**

**Supplementary Figure 2. Response frequencies according to whether respondents can usually work from home or can never/rarely work from home**
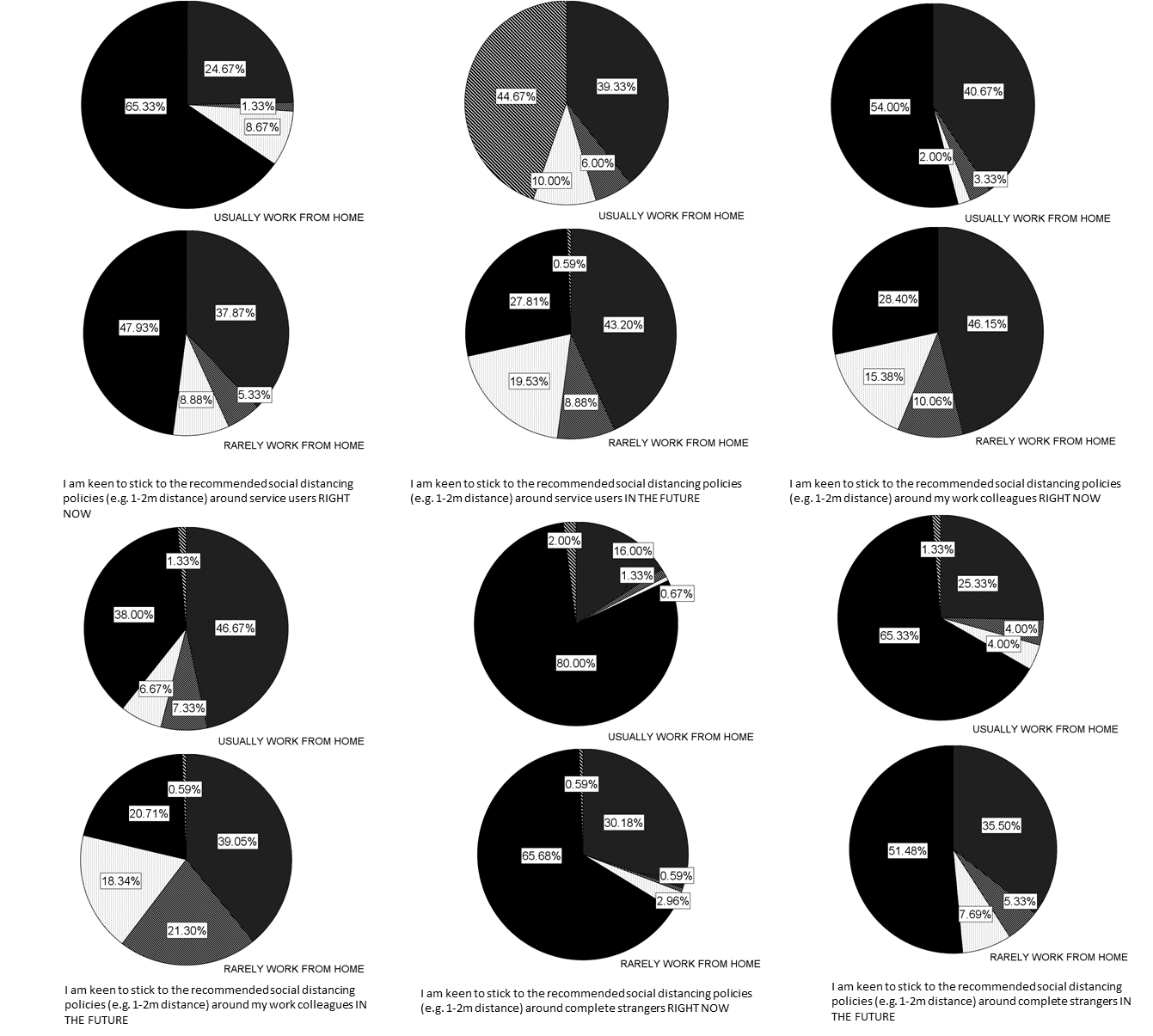

Supplement: Supplementary file 1 — Figure S1. Response frequencies according to whether job role involves patient contact. Figure S2. Response frequencies according to whether respondents can usually work from home or can never/rarely work from home. [file INM-30-1366-s001.docx]
